# Caspase 8 gene variants in healthy North Indian population and comparison with worldwide ethnic group variations

**DOI:** 10.4103/0971-6866.73406

**Published:** 2010

**Authors:** Ginu P. George, Rama D. Mittal

**Affiliations:** Department of Urology and Renal Transplantation, Sanjay Gandhi Post Graduate Institute of Medical Sciences, Raebareli Road, Lucknow - 226 014, Uttar Pradesh, India

**Keywords:** Caspase, ethnicity, haplotype, PCR-RFLP

## Abstract

**BACKGROUND:**

Many strategies are being used for the quest for the disease causing genes. Inter-individual variations in several genes exist. Thus, even if they share the same disease-associated allele, the genomic backgrounds – and hence potential interacting alleles at other loci – of people with different regional ancestries may differ, with a consequent variation in the severity of their disease.

**MATERIALS AND METHOD:**

The present study was conducted to determine the distribution of Caspase 8 IVS12-19G/A, Caspase 8D302H, Caspase 8 -652del and Caspase 8 -678del polymorphisms (as frequency distribution of caspases in Indians generally is not yet known), which was then compared with different populations globally. Polymerase chain reaction (PCR)-based analysis was conducted in 205 normal healthy individuals of similar ethnicity.

**RESULTS:**

The variant allele frequencies were 17.6% (A) in Caspase 8 IVS12-19G/A, 13.2% (H) in Caspase 8D302H, 23.2% (Del) in Caspase 8 -652del and 24.6% (Del) in Caspase 8 -678del. Further, comparison of frequency distribution of these genes was done with various published studies of different ethnic groups globally.

**CONCLUSION:**

It is anticipated from our results that the frequency of these caspase genes exhibits distinctive patterns in India, which could perhaps be attributed to ethnic variation. This study is important as it can form a baseline for screening individuals who are at high risk due to exposure to environmental carcinogens and cancer predisposition, and therefore, might help in investigating linked polymorphisms in a way that will not obscure potential associations between genotype and phenotype.

## Introduction

In the past decade, the number of publications on caspases has snowballed from zero to more than 10,000. The critical roles of caspases in regulating signaling and execution of apoptosis are well accepted. Caspase activation has been demonstrated in many diseases characterized by abnormal cell death. Apoptosis is a major mechanism aimed at ensuring proper development and organism homeostasis. Due to its role in the elimination of virally infected and damaged cells, apoptosis also plays a central role in the prevention of diseases. The enormous power of this process, however, makes the dysregulation of apoptosis deleterious for the organism and can lead to a host of pathologies.[1] The induction of apoptosis in these diseases may significantly contribute to their pathogenesis. In addition to their roles in apoptosis, multiple caspases possess additional functions not related to the cell death, which may or may not involve other components of the cell death machinery. Caspase 8 (CASP8) is a key regulator of apoptosis or programmed cell death, an essential defense mechanism against hyperproliferation and malignancy. The properties of caspase 8 are very well characterized and its role as an apical caspase in death receptor signaling has been well established through biochemical, molecular and cell biology studies.

Single-nucleotide polymorphisms (SNPs) are the most common form of human genetic variation and may contribute to an individual’s susceptibility to cancer. Some variants in apoptosis pathway genes are associated with the susceptibility to human cancers.[2] Functional SNPs in genes involved in apoptotic pathways may modulate cellular apoptotic capacity in response to DNA damage. Resistance to apoptosis or reduced cellular apoptotic capacity provides a survival advantage of the cells that may develop into cancer cells, commonly seen in almost all types of malignant diseases, and mutations in the genes involved in apoptotic pathways are one of the molecular mechanisms underlying carcinogenesis and cancer therapy.[3–5] It is likely that the efficiency of these apoptotic pathways is genetically determined because functional polymorphisms in genes involved in these apoptotic pathways may modulate the phenotype, thus contributing to individual variation. In recent years, genetic variants in caspase mediated apoptosis and their role in human cancer susceptibility have been getting more and more attention, especially the apoptosis initiator caspase 8.[6] Therefore, this study was undertaken to test *Caspase 8 IVS12-19 G>A, Caspase 8D302H G>C, Caspase 8 -652 6Ndel* and *Caspase 8 -678_-673del* polymorphisms in North Indian healthy population. Moreover, we have also compared these polymorphisms with different ethnic groups worldwide because the genetic polymorphisms often vary between different ethnic groups.

## Materials and Methods

### Subjects

Healthy and genetically unrelated individuals either visiting the hospital or health awareness camps for a routine checkup, or healthy hospital employees were recruited as the controls (*n* = 205). All the controls were age- and sex-matched with similar ethnicity and had no evidence of malignancy or chronic disease. The mean age of the controls was 66.0 ± 7.4 years. The participation rate was 100%, and blood samples were drawn from all the subjects. An epidemiologic questionnaire was designed to collect data on demographic characteristics such as smoking and occupation history, lifestyle factors etc. from the study participants. At the end of the interview, a 5-ml blood sample was drawn into coded tubes. Informed and written consent was taken from all the subjects when interviewing for the demographic details and blood sample collection. The Ethical Review Board of the Institute approved the study.

We examined the association of four polymorphic sites of caspase 8 gene (*Caspase 8 IVS12-19G/A, Caspase 8D302H, Caspase 8 -652del* and *Caspase 8 -678del*) and reviewed adequate number of epidemiologic studies of different ethnicities on caspase to conduct a comparative analysis for genetic polymorphisms in apoptotic genes, focusing on caspase 8 SNPs.

### DNA extraction

Five milliliters of blood was collected in ethylenediamine tetraacetic acid (EDTA) vials and DNA was extracted from blood lymphocytes using “salting out” method.[7]

### Genotyping

All the study samples were genotyped for four SNPs in caspase genes that included *Caspase 8 IVS12-19 G>A, Caspase 8D302H G>C, Caspase 8 -652 6Ndel* and *Caspase 8 -678_-673del*, using Polymerase Chain Reaction- Restriction Fragment Length Polymorphism (PCR-RFLP) for the first two SNPs and the other two were insertion/deletion polymorphisms.

### Prevalence of gene variants

MEDLINE search for *Caspase 8 IVS12-19 G>A, Caspase 8D302H G>C, Caspase 8 -652 6Ndel* and *Caspase 8 -678_-673del* and “polymorphism” was done for articles published after 2001. The search was limited to human subjects. Studies that reported only allele frequencies and no genotype frequencies were not included. Studies based on fewer than 90 persons were excluded. When more than one article was identified for the same study population, we included the most recent publication. We identified two publications reporting on the prevalence of *Caspase 8 IVS12-19G>A* polymorphism,[6,8] four publications on *Caspase 8D302H G>C*,[2,9,10] three studies on *Caspase 8 -652 6Ndel*[11–13] and only one publication for *Caspase 8 -678_-673del*,[8] which were subsequently used for comparing with our study.

### Statistical analysis

The genotype and allelic frequencies of different populations were compared by Pearson’s χ^2^ test, using the computer software SPSS for windows (version 11.5). Court-Lab (web-based software) was used to examine Hardy–Weinberg equilibrium (www.tufts.edu). *P* value <0.05 was considered statistically significant.

## Results

Table 1 shows the genotypes and allele frequency distribution of *Caspase 8 IVS12-19 G>A, Caspase 8D302H G>C, Caspase 8 -652 6Ndel* and *Caspase 8 -678_-673del* polymorphisms in North Indian population. The genotype distributions in DNA of healthy individuals of all the selected polymorphic sites in four genes were in agreement with Hardy–Weinberg equilibrium. The frequency distribution of different genotypes and alleles of these four polymorphic sites in different populations with reference to ours were compared [Table 2] using χ^2^ tests. The minor variant allele frequency in our population was as follows: 17.6% for *Caspase 8 IVS12-19G/A*, 13.2% for *Caspase 8D302H*, 23.2% for *Caspase 8 -652del* and 24.6% for *Caspase 8 -678del*.

**Table 1:** Genotypes and allele frequency distribution of CASP8 IVS12-19G/A, CASP8 D302H, CASP -652del and CASP8 -678del gene polymorphism in healthy individuals of North India

Gene	Genotype	Observed (%)	Expected (%)	Minor allele frequency	*P*-value (HWE)
CASP8 IVS12-19G/A	GG	70.7	67.9		0.05
	GA	23.4	29.0		
	AA	5.9	3.1	17.6	
CASP8 D302H	DD	75.6	75.3		
	DH	22.4	22.9		0.82
	HH	2.0	1.7	13.2	
CASP8 652del	I/I	56.6	59.1		0.16
	I/D	40.5	35.6		
	D/D	2.9	5.4	23.2	
CASP8 678del	I/I	55.6	56.8		0.52
	I/D	39.5	37.1		
	D/D	4.9	6.1	24.6	

**Table 2:** Genotypes and allele frequency distribution of CASP8 IVS12-19G/A, CASP8 D302H, CASP -652del and CASP8 -678del gene polymorphisms in various populations and *P* values in comparison to North Indian population

Country/ethnicity	n	Mean age ± SD/range		Genotype		*P* value	Minor allele frequency	Reference
CASP8 IVS12-19G/A			GG	GA	AA	A		
North India	205	66.0 ± 7.4	145 (70.7)	48 (23.4)	12 (5.9)	Ref	17.6	Present Study
Korea	432	60.9 ± 9.3	226 (52.6)	166 (38.4)	40 (9.3)	0.02	28.5	Son *et al*.[8]
Chinese	838	59.5 ± 10.9	433 (51.7)	335 (40)	70 (8.4)	0.04	28.3	Liu *et al*.[6]
CASP8 D302H			DD	DH	HH	H		
North India	205	66.0 ± 7.4	155 (75.6)	46 (22.4)	4 (2)	Ref	13.2	Present Study
Sheffield	964	45–80	675 (70)	265 (27.5)	24 (2.5)	0.55	16	MacPherson *et al*.[2]
East Anglia	2082	45–81	1591 (76.4)	450 (21.6)	4 (2)	0.99	13	MacPherson *et al*.[2]
Non-Hispanic	835	—	615 (73.7)	207 (24.8)	13 (1.6)	0.73	14	Li *et al*.[9]
US	170	57.9 ± 12.1	127 (74.7)	38 (22.4)	5 (2.9)	0.23	14.1	Hu *et al*.[10]
CASP -652del			I/I	I/D	D/D	D		
North India	205	66.0 ± 7.4	116 (56.6)	83 (40.5)	6 (2.9)	Ref	23.2	Present Study
German	1039	18–68	270 (26)	506 (48.7)	263 (25.3)	<0.001	49.7	Frank *et al*.[11]
Chinese	368	—	205 (55.7)	138 (37.5)	25 (6.8)	0.06	25.5	Wang *et al*.[12]
Polish	965	—	274 (28.4)	499 (51.7)	192 (19.9)	<0.001	51	Cybulski *et al*.[13]
CASP8 -678del			I/I	I/D	D/D	D		
North India	205	66.0 ± 7.4	114 (55.6)	81 (39.5)	10 (4.9)	Ref	24.6	Present Study
Korea	432	60.9 ± 9.3	249 (57.6)	161 (37.3)	22 (5.1)	0.98	23.7	Son *et al*.[8]

In the case of *Caspase 8 IVS12-19G/A*, significant frequency distribution was observed in Korea and China as compared to our population. Genotype and allele distribution of *Caspase 8D302H* polymorphism was also different from that of Sheffield, East Anglia, USA and Non-Hispanics as compared to our population. *Caspase -652del* frequency distribution also varied in German, Chinese and Polish population in comparison to our study. Study of *Caspase 8 -678del* polymorphism is not common worldwide; only a few studies have been reported. Based on reports, a different pattern of *Caspase 8 -678del* polymorphism and allele frequency was reported only in the Koreans.

A major significant difference was observed in case of *Caspase 8 IVS12-19G/A* in Korean and Chinese population (*P* = 0.02 and 0.04, respectively), and also in case of *Caspase -652del* of German and Polish ethnicity (*P* < 0.001 for both).

## Discussion

Several candidate polymorphisms in the caspase 8 gene have been reported recently in public databases (http://www.ncbi.nlm.nih.gov/SNP). So, some of these SNPs in the genome, with a high degree of variability make these informative genetic markers useful for disease susceptibility. Therefore, these variants may influence CASP-8 activity, thereby modulating susceptibility. Genetic polymorphisms in the caspase genes may influence cancer risk by altering expression levels and functions of these genes. In recent years, genetic variants in caspase mediated apoptosis and their role in human cancer susceptibility have been getting more and more attention, especially the apoptosis initiator caspase 8. Due to marked differences in the distribution of caspase gene polymorphisms between various ethnicities, the data from “normal healthy” populations are of special interest for the adequate evaluation of the relevance of the investigated genetic markers in susceptibility, manifestation, prognosis or treatment of diseases. However, it is noteworthy to conduct extensive investigations about the distribution of these genes in different ethnic groups.

The variation in our Indian population from other world population signifies the impact of ethnicity. It is well recognized that ethnic background may influence the susceptibility to certain diseases.[14] Due to the different socio-cultural traditions, Indian population is believed to be most diverse. Future implications for preventive and early intervention strategies in cancer can therefore be accomplished by the study of genetic variation, which can elucidate critical determinants in environmental exposure and cancer. The differences in allele frequencies detected among these studies might be due to ethnic variation, heterogeneity of study populations and/or different sample sizes, as there have been very different selective pressures driving different disease susceptibilities in genetically heterogeneous populations.[15,16]

In the *CASP 8 IVS12-19G/A* polymorphism, the (A) allele frequency in Indian population was 17.6%, whereas it was significantly higher in Korean and Chinese subjects (28.5 and 28.3%, respectively). The (H) allele frequency in *CASP D302H* polymorphism was 13.2% in our population. No significant difference was observed in the populations from Sheffield, East Anglia, among the Non-Hispanics and from USA. In *CASP -652del* polymorphism, the (Del) allele frequency in Indian population was 23.2%, which was significantly different and higher in German and Polish subjects while it was the same in China. On the other hand, the allele frequency for *CASP8 -678del*, (Del) allele was 24.6 which was the same as in Korean subjects.

The minor variant allele frequencies of Sheffield, East Anglia, Non-Hispanics and USA were found to be almost similar to that of our northern population for *CASP 8 D302H* (13.2% vs. 16%, 13%, 14% and 14.1%, respectively), a similar pattern was observed in case of *CASP 8 -678del* (24.6% vs. 23.7%) in our population and in a study by Son *et al*. (2006) in Korean population This suggested that gene variants were unaffected based on geographic location. Unlinked genes undergo more or less independent changes in allele frequency; this produces geographically cross-cutting allele frequency clines found among genes.[14]

One way to determine what is driving health disparities is to have more focused genetic research within specific identifiable populations whose unusually high (or low) risk or population history makes them advantageous for finding susceptibility genes.[17] But this raises the potential danger that the disease might be racialized using the new genetic information.[18–20] The advantage of such kind of study may form the basis for future establishment of epidemiologic and clinical databases. Population differences in disease incidence and mortality are due to a range of genetic and socio-cultural factors. Health disparities among populations are well documented, yet poorly understood. Susceptibility genotypes are shared across our species, race or equivalent socio-culturally defined measures are often highly correlated with socioeconomic or other environmental or lifestyle factors. The same genotype may have very different risks in different environments, and this can mean in different populations; secular trends for most major diseases show clearly that this is the typical case.[14]

Therefore, our observation suggests that CASP 8 polymorphisms with their variants could be used as biomarkers for disease susceptibility and may be used for assessing the risk of cancer development. A single larger study with thousands of subjects and tissue-specific biochemical and biological characterization is warranted to further evaluate potential gene-to-gene and gene-to-environment interactions on CASP gene polymorphisms and cancer risk. The differences in the distribution of these genes between North Indian healthy population and other ethnic groups may help in building a silhouette that would help in assessing the disease predisposition and prevalence. More emphasis is needed on evaluating polymorphisms, alone or in combinations, as modifiers of risk from relevant environmental/lifestyle exposures.
